# Association of the Polygenic Risk Score With the Probability of Prodromal Parkinson’s Disease in Older Adults

**DOI:** 10.3389/fnmol.2021.739571

**Published:** 2021-12-21

**Authors:** Maria I. Maraki, Alexandros Hatzimanolis, Niki Mourtzi, Leonidas Stefanis, Mary Yannakoulia, Mary H. Kosmidis, Efthimios Dardiotis, Georgios M. Hadjigeorgiou, Paraskevi Sakka, Alfredo Ramirez, Benjamin Grenier-Boley, Jean-Charles Lambert, Stefanie Heilmann-Heimbach, Maria Stamelou, Nikolaos Scarmeas, Georgia Xiromerisiou

**Affiliations:** ^1^Section of Sport Medicine and Biology of Exercise, School of Physical Education and Sport Science, National and Kapodistrian University of Athens, Athens, Greece; ^2^Department of Nutrition and Dietetics, School of Health Sciences, Hellenic Mediterranean University, Crete, Greece; ^3^Department of Psychiatry, National and Kapodistrian University of Athens Medical School, Eginition Hospital, Athens, Greece; ^4^Neurobiology Research Institute, Theodor-Theohari Cozzika Foundation, Athens, Greece; ^5^First Department of Neurology, Eginition Hospital, National and Kapodistrian University of Athens Medical School, Athens, Greece; ^6^Center of Clinical, Experimental Surgery and Translational Research, Biomedical Research Foundation of the Academy of Athens, Athens, Greece; ^7^Laboratory of Cognitive Neuroscience, School of Psychology, Aristotle University of Thessaloniki, Thessaloniki, Greece; ^8^School of Medicine, University of Thessaly, Larissa, Greece; ^9^Department of Neurology, Medical School, University of Cyprus, Nicosia, Cyprus; ^10^Athens Association of Alzheimer’s Disease and Related Disorders, Marousi, Greece; ^11^Division of Neurogenetics and Molecular Psychiatry, Department of Psychiatry and Psychotherapy, Faculty of Medicine and University Hospital Cologne, University of Cologne, Cologne, Germany; ^12^Department of Neurodegenerative Diseases and Geriatric Psychiatry, University Hospital Bonn, Bonn, Germany; ^13^German Center for Neurodegenerative Diseases (DZNE Bonn), Bonn, Germany; ^14^Department of Psychiatry and Glenn Biggs Institute for Alzheimer’s and Neurodegenerative Diseases, San Antonio, TX, United States; ^15^Cluster of Excellence Cellular Stress Responses in Aging-Associated Diseases (CECAD), University of Cologne, Cologne, Germany; ^16^INSERM, CHU Lille, Institut Pasteur de Lille, U1167-RID-AGE Facteurs de Risque et Determinants Moléculaires des Maladies Liées au Vieillissement, University of Lille, Lille, France; ^17^Institute of Human Genetics, School of Medicine and University Hospital Bonn, University of Bonn, Bonn, Germany; ^18^Parkinson’s Disease and Movement Disorders Department, HYGEIA Hospital, Athens, Greece; ^19^Taub Institute for Research in Alzheimer’s Disease and the Aging Brain, The Gertrude H. Sergievsky Center, Department of Neurology, Columbia University, New York, NY, United States

**Keywords:** genetics, Parkinsonism, elderly, neurodegeneration, cognition

## Abstract

Several studies have investigated the association of the Parkinson’s disease (PD) polygenic risk score (PRS) with several aspects of well-established PD. We sought to evaluate the association of PRS with the prodromal stage of PD. We calculated PRS in a longitudinal sample (*n* = 1120) of community dwelling individuals ≥ 65 years from the HELIAD (The Hellenic Longitudinal Investigation of Aging and Diet) study in order to evaluate the association of this score with the probability of prodromal PD or any of the established risk and prodromal markers in MDS research criteria, using regression multi-adjusted models. Increases in PRS estimated from GWAS summary statistics’ ninety top SNPS with *p* < 5 × 10^–8^ was associated with increased odds of having probable/possible prodromal PD (i.e., ≥ 30% probability, OR = 1.033, 95%CI: 1.009–1.057 *p* = 0.006). From the prodromal PD risk markers, significant association was found between PRS and global cognitive deficit exclusively (*p* = 0.003). To our knowledge, our study is the first population based study investigating the association between PRS scores and prodromal markers of Parkinson’s disease. Our results suggest a strong relationship between the accumulation of many common genetic variants, as measured by PRS, and cognitive deficits.

## Introduction

Currently, several non-motor symptoms have been associated with an increased risk to develop Parkinson’s disease (PD) in otherwise healthy individuals, while ongoing research aims to validate a variety of candidate PD biomarkers based on imaging, genetic, proteomic, or metabolomic signatures, supplemented by work on tissue markers accessible to minimally invasive biopsies. In fact, the recently defined MDS research criteria for prodromal PD include a combination of risk and prodromal markers, in an effort to define target populations of future disease modification trials (Heinzel et al., 2019a). Critically, these criteria have been prospectively validated in six independent cohort studies (Fereshtehnejad et al., 2017; Pilotto et al., 2017; Mahlknecht et al., 2018; Mirelman et al., 2018; Giagkou et al., 2020).

Additionally, genetic markers have been integrated in the MDS criteria for prodromal PD in order to improve the accuracy of prodromal PD diagnosis (Heinzel et al., 2019a). Individuals with rare high-penetrance genetic mutations are considered a distinct subgroup with a specific risk according to the mutation. For intermediate strength genetic factors, such as mutations in GBA and LRRK2, the prodromal PD risk is age-dependent and can be calculated, based on the penetrance of mutation and the PD risk, across different age groups. However, the cumulative predictive effect of common and low individual effect strength genetic risk variants has been recently introduced in the criteria. The aforementioned variable, PRS, has been introduced and likelihood-ratio (LR) is estimated according to its value (Heinzel et al., 2019a).

More specifically, polygenic score association analysis examines if a polygenic score, calculated as the cumulative risk of several small effect alleles detected by genome-wide association study (GWAS), confers a high risk for a disease and if the same sets of risk alleles are shared between cohorts/data sets. An influential role for polygenic inheritance in PD is strongly supported by a number of studies, that revealed a significant association between disease risk, age of onset, motor progression, and cognitive decline with polygenic risk scores (PRS), calculated from GWAS summary statistics for PD (Nalls et al., 2019).

However, all studies published today, examined the association of PRS with several aspects of well-established PD. There are no studies that investigate the association of this score with the probability of prodromal PD and its risk markers. In this study, we calculated PRS in a longitudinal sample of community dwelling individuals ≥ 65 years from the HELIAD study (The Hellenic Longitudinal Investigation of Aging and Diet) to evaluate the association of this score with the probability of prodromal PD. We further investigated whether there is an association between any of the established risk and prodromal markers in MDS criteria with PRS. The identification of the genetic influence on prodromal phase will allow the detection of a pure association between multiple domains of the disorder and PRS without the interaction of other clinical features.

## Materials and Methods

### Study Population

The Hellenic Longitudinal Investigation of Aging and Diet (HELIAD) is a large-scale, population-based, multidisciplinary study designed to assess the prevalence, incidence, and risk factors of neuropsychiatric conditions of aging in Greece (Dardiotis et al., 2014). We randomly selected participants among community-dwelling individuals from two areas in Greece (age ≥ 65, no exclusion criteria), and qualified neurologists and other health professionals collected demographic, medical, environmental, neuropsychological, and lifestyle information. Senior or junior neurologists examined all participants. Details on HELIAD design, participation rates and clinical and neuropsychological evaluation have been previously published (Dardiotis et al., 2014; Ntanasi et al., 2017, 2020; Tsapanou et al., 2017; Kosmidis et al., 2018; Bougea et al., 2019; Maraki et al., 2019a,b; Giagkou et al., 2020). For the present study, we excluded from the analysis those participants who were diagnosed with PD or dementia with Lewy bodies (DLB) and participants for whom the presence or absence of these diagnoses could not be ascertained (Maraki et al., 2019b; Giagkou et al., 2020). The study protocol was approved by the relevant institutional review boards (University of Thessaly, Larisa, Greece, and the National and Kapodistrian University of Athens, Greece, Ethics Committees). All participants or authorized representatives gave their written informed consent prior to participation.

### Clinical and Other Assessments

We collected information regarding sociodemographics (e.g., gender, age, years of education, number of vehicles, home size, rental vs. ownership status, type and amount of tobacco use). We used a structured pretested questionnaire to evaluate pesticide exposure and a semiquantitative food frequency questionnaire to evaluate coffee consumption. Physical inactivity was ascertained *via* a leisure activities structured questionnaire. We also collected information regarding medical and neurological conditions, neuropsychiatric symptoms, current medications, hospitalizations, and injuries of the participants, and information about the medical/neurological histories of the participants’ first-degree relatives. In addition, we conducted an extensive structured physical evaluation of the participant, including neurological signs and symptoms. We used the 15-item Geriatric Depression Scale (Fountoulakis et al., 1999) and the 7-item anxiety subscale of the Hospital Anxiety and Depression Scale (Michopoulos et al., 2008) to screen for depressive symptoms and anxiety during the preceding week, respectively. We used the 12-item Medical Outcomes Study Sleep Scale (Hays et al., 2005) to assess the quantitative and qualitative features of sleep during the preceding month and the Blessed Dementia Scale (Blessed et al., 1968) for the perceived changes in performance of daily activities and self-care habits. Parkinsonian signs and symptoms were evaluated using the UPDRSIII (Fahn and Elton, 1987). We also administered a structured questionnaire to determine whether core (e.g., Parkinsonism), suggestive [e.g., REM sleep behavior disorder (RBD)] or supportive features (e.g., systematized delusions) of the revised diagnostic criteria for Dementia with Lewy bodies were present (McKeith et al., 2005; Anastasiou et al., 2017). We also administered the 12-item Neuropsychiatric Inventory (Cummings, 1997). The information obtained was reviewed, and the clinical diagnosis of each participant was reached using published criteria at expert consensus meetings.

### Calculation of the Probability of pPD

We collected data for the vast majority of 2019 MDS pPD markers (Berg et al., 2015; Heinzel et al., 2019b): 16 of 21 (seven out of the 10 risk markers and nine out of the 11 prodromal markers) (Maraki et al., 2019b; Giagkou et al., 2020). In more details, the *risk* markers assessed were: sex, pesticide exposure, non-use of caffeine, non-smoking status, presence of 1**st** degree relative with PD, Type 2 Diabetes mellitus (DM2) and physical inactivity. Information on occupational solvent exposure, substantia nigra hyperechogenicity and plasma urate were not available. The *prodromal* markers assessed were: possible RBD, subthreshold parkinsonism, constipation, excessive daytime somnolence, symptomatic orthostatic hypotension, erectile dysfunction, urinary dysfunction, depression or anxiety without depression and global cognitive deficit. Information on olfactory dysfunction and tracer uptake of the presynaptic dopaminergic system (single-photon emission computed tomography or positron emission tomography) were not available. As MDS and recent studies have suggested, pPD probability may be calculated using available markers in each cohort, although may be underestimated when markers are limited (Mahlknecht et al., 2016; Skorvanek et al., 2017). Details on how we evaluated the above risk and prodromal markers can be found in previous publications on the probability of pPD in HELIAD population (Bougea et al., 2019; Maraki et al., 2019a,b; Giagkou et al., 2020; Ntanasi et al., 2020).

We calculated the pPD probability for non-PD/DLB participants, according to MDS guidance (Berg et al., 2015; Mahlknecht et al., 2016; Heinzel et al., 2019b), as previously reported (Bougea et al., 2019; Maraki et al., 2019a,b; Giagkou et al., 2020; Ntanasi et al., 2020). In more detail, we determined prior (pretest) pPD probability according to the participant’s age. We calculated the individualized likelihood ratios (LRs) for every risk and prodromal marker; missing values were scored 1.0. We then computed the total risk LR and total prodromal LR separately by multiplying the corresponding markers. Next, these LRs were multiplied to provide the total LR. Then, we calculated the final posttest pPD probability by combining pretest probability with total LR. For the purpose of the present analysis, we used posttest pPD probability as a continuous variable, but also the cut-offs of 30% (possible/probable pPD), 50% and 80% (probable pPD) (Berg et al., 2014, 2015).

### Genotyping and Imputation

Genome-wide genotyping was performed for 1,446 individuals in three different centers, at the “Centre National de Recherché en Génétique Humaine” (GNRGH, Evry, France), at Life and Braincenter (Bonn, Germany) and at the Erasmus Medical University (Rotterdam, Netherlands) using the Illumina Infinium Global Screening Array (GSA, GSAsharedCUSTOM_24 + v1.0), as part of the European Alzheimer DNA biobank (EADB) project. Base calling of the raw reads was performed at CNRGH. A detailed description of the EADB genotyping, QC and imputation can be found elsewhere. In summary, variants included in the marker list for removal, provided by Illumina, or variants not uniquely aligned in GRCh37 genome were excluded for further analysis. Moreover, variant intensity quality control (QC), was conducted for all autosomal variants, according to established thresholds, while sex-check was also performed using chromosome X variants (Grove et al., 2013).

Next, we performed sample quality control using PLINK v1.9 software (Purcell et al., 2007; Chang et al., 2015; Chang, 2020). Specifically, samples with missingness > 0.05, sex inconsistencies or with heterozygosity rate that deviated more than ± 6 SD from the mean, were excluded. To identify population outliers, we run Principal Component Analysis (PCA), using as reference dataset the population of 1000 Genome (phase 3) and we projected the combined dataset (1000 GP3 samples and the EADB samples) onto two dimensions, using the flashPCA2 software (Abraham et al., 2017). To control for cryptic relatedness, we excluded individuals with a kinship coefficient more than 0.125 (cut-off for third-degree relatives), yielding a final sample size of 1,120 unrelated individuals.

Regarding quality controls of variants, we excluded variants showing a missingness > 0.05 in at least one genotyping center or having a differential missingness test *P* < 10**^–10^**. The Hardy-Weinberg equilibrium test (*p* < 5e**^–8^**) was performed only in controls and for each genotyping center/country separately.

To improve the accuracy of imputation, we compared the frequencies of variants (chi-square test) against two reference panels, the population of the Haplotype Reference Consortium r1.1 (HRC) (McCarthy et al., 2016), excluding samples from 1,000 genomes as well as the Finnish and the non-Finnish population of Genome Aggregation Database v3 (gnomAD) (Karczewski et al., 2020). Variants showing a x2 > 3,000 in both HRC and gnomAD or a x2 > 3,000 in one reference panel and not present in the other were excluded. Finally, GWASs were performed between controls across genotyping centers to assess frequency differences between genotyping centers, using the software SNPTEST (Marchini et al., 2007), under an additive model and adjusting on associated Principal Components (PCs). Variants having a Likelihood Ratio Test of *p* < 10**^–5^** were excluded. Furthermore, we removed ambiguous variants with Minor Allele Frequency (MAF) > 0.4 and we kept only one copy of any duplicated variants, prioritizing the one with the lowest missingness.

All samples and variants, passing the above QC metrics were imputed o Michigan Imputation Server (v1.2.4) (Das et al., 2016), using the TOPMed Freeze 5 reference panel. Phasing and imputation were performed using EAGLE v2.4 and Minimac4 v4-1.0.2 software, respectively.

### Polygenic Risk Score Calculation

Imputed dosages for a total of 5,611,082 SNPs with MAF > 0.05, call rate > 95% and imputation quality score > 0.4 were converted to best-guess genotypes for PRS computation. The PRSice software^1^ (Choi and O’Reilly, 2019) was utilized to construct PRSs for each individual applying the clumping and thresholding (C + T) method, following the approach originally described by the International Schizophrenia Consortium (International Schizophrenia et al., 2009). Separate PRS for Parkinson’s disease were computed, as the weighted sum of the risk increasing alleles that each individual carries at each SNP locus multiplied by the effect size for the reference allele on the basis of large-scale genome-wide association (GWAS) meta-analysis summary data (i.e., discovery samples) (International Schizophrenia et al., 2009). Different sets of SNPs were filtered in the HELIAD sample (i.e., target sample) by applying increasing *p-*value thresholds to the discovery GWAS summary statistics and appropriate linkage disequilibrium (LD)-based SNP clumping (SNP with r**^2^** > 0.1 in 250 kb-windows were removed) was performed to ensure that only independent markers are included in the computed PRS. Markers within the major histocompatibility complex (MHC) LD region on chromosome 6 (hg19; chr6:27–33 Mb) were also excluded from PRS computation process due to the high polymorphic nature of this region.

For each subject, we computed different genome-wide PRSs based on *a priori* set of eight *P*-value thresholds (PT) (i.e., 1e-4, 0.001, 0.05, 0.1, 0.2, 0.3, 0.4, 0.5), to identify the best threshold for predicting the outcomes of interest.

### Statistical Analysis

HELIAD participants with available data on PRS and probability of pPD were included in the present analysis. Normality of data was graphically explored using Q-Q plots. Values are presented as means ± SD or medians (Q1, Q3) for continuous, normally and not normally distributed, respectively, and as frequencies (%) for categorical variables. Differences between two groups (e.g., possible vs. not possible pPD, i.e., ≥ vs. < 30% pPD probability etc.) were tested by unpaired *t*-test or Mann–Whitney rank tests for normally and not normally distributed continuous variables, respectively, and Chi-Square tests for categorical variables. Differences between PRS quartiles were tested with one-way ANOVA followed by *post hoc* Student *t*-tests, or Kruskal–Wallis followed by Mann–Whitney rank tests, for normally and not normally distributed continuous, respectively, and Chi-Square tests for categorical variables.

The associations between PRS and probability of pPD (log-transformed data) were evaluated with linear regression analyses or logistic regression analyses [when probability of pPD was treated as dichotomous variable (i.e., ≥ 30% etc., probability of pPD)]. Furthermore, we used linear regression models to investigate relations between PRS and total LR for prodromal or risk markers (log-transformed data). We also used logistic regression analyses to investigate relations between PRS and each prodromal marker. All models were adjusted for MDSC1, MDSC2, age and sex. The PRS was entered into the models both as a continuous variable, as well as quartiles (comparing the fourth-higher *vs*. other quartiles). We did not adjust for age and/or sex when the dependent variable (e.g., probability of pPD) was calculated using age and/or sex among others (see above: calculation of pPD probability). Nevertheless, on a purely exploratory attempt, we calculated supplementary models adjusting for age and sex.

Reported *p*-values are nominal. Statistical significance was set at *p* ≤ 0.05. All data were analyzed using SPSS statistical software (SPSS 19.0, SPSS Inc., United States).

### Sensitivity Analysis

In an effort to distinguish the association of PRS with cognitive dysfunction in the sole context of pPD we performed additional analysis excluding participants with dementia (*n* = 39) and those with mild cognitive impairment (MCI, *n* = 143). The diagnosis of dementia was based on Diagnostic and Statistical Manual of Mental Disorders -IV-text revision criteria [American Psychiatric Association [APA], 2000] while MCI was diagnosed according to Petersen Criteria (Petersen et al., 2018).

## Results

### Genotyping and Subjects Characteristics

European ancestry of the cohort samples was confirmed with principal component analysis, as all samples clustered accordingly with European samples from the 1,000 G dataset. We excluded third degree relatives with pihat scores > 0.125 (*n* = 130).

Non-PD/DLB participants were included in our analysis and their main characteristics are shown in Table 1. Most participants (745, 66.5%) had less than 5% probability of pPD, while 5.3% of the sample had possible or probable pPD, i.e., 30% or more pPD probability (Table 1).

**TABLE 1:** Sample characteristics (*n* = 1,120).

Characteristics	All
**Sex, *n* (%)**	
Male	475 (42.4%)
Female	645 (57.6%)
Age (years), mean ± SD	74 ± 5
Years of education, median (Q1, Q3)	6 (4, 9)
Job Type, *n* (%)	
Manual labor	782 (74.9%)
Mental labor	262 (25.1%)
**Socioeconomic Status, *n* (%)**	
Lower	542 (48.4%)
Higher	578 (51.6%)
PRS, *mean* ± SD	−1.60 ± 12.61
Probability of pPD, *median* (Q1, Q3)	2.74 (1.14, 7.08)
≥80% probability of pPD, *n* (%)	11 (1.0%)
≥50% probability of pPD, *n* (%)	28 (2.5%)
≥30% probability of pPD, *n* (%)	59 (5.3%)

### Association of the PRSs With the Probability of pPD

**FIGURE 1:**
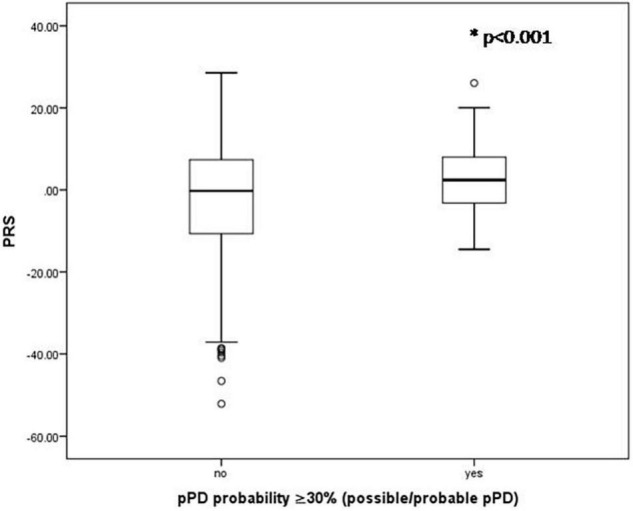
Boxplots of PRS for those having or not 30% or more pPD probability. *P*-value derived from unpaired *t*-test.

**FIGURE 2:**
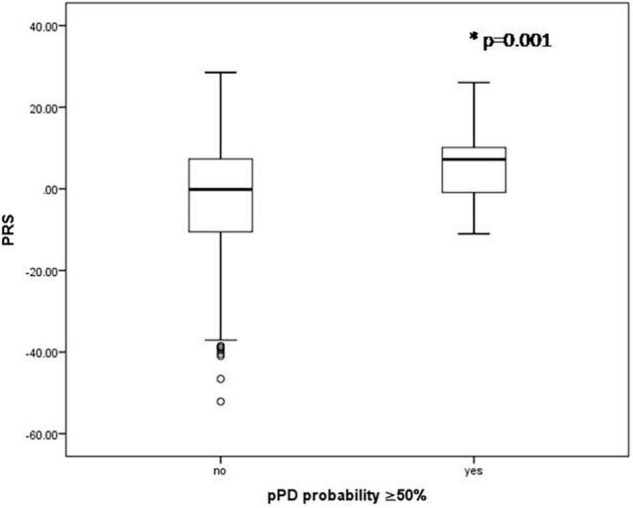
Boxplots of PRS for those having or not 50% or more pPD probability. *P*-value derived from unpaired *t*-test.

There were no significant associations between any *p*_*T*_ cutoffs (*p*_*T*_: significance level probability value thresholds for SNP selection in the discovery sample) and the probability of pPD (*p* > 0.05). Additionally, for most *p*_*T*_ cutoffs there was no association between PRS and total LR for risk markers (*p* > 0.05) as well as total LR for prodromal markers (*p* > 0.05). However, PRS estimated from 90 top SNPS with *p* < 5 × 10^–8^ was significantly higher in the possible/probable pPD group (i.e., in those with 30% or more pPD probability) (2.83 ± 8.85 vs. −1.85 ± 12.75, *p* < 0.001; Figure 1) and in those with 50% or more pPD probability (5.09 ± 9.23 vs. −1.77 ± 12.65, *p* = 0.001; Figure 2). Logistic regression models adjusted for MDSC1 and MDSC2 showed that increase in PRS was associated with increased odds of having 30 or 50% or more pPD probability (OR = 1.033, 95%CI: 1.009–1.057, *p* = 0.006; OR = 1.052, 95%CI: 1.015–1.089, *p* = 0.005), while further adjustment for age and sex did not change the results (OR = 1.036, 95%CI: 1.011–1.060, *p* = 0.004 and OR = 1.056, 95%CI: 1.019–1.096, *p* = 0.003, respectively).

### Correlations Between PRSs and Prodromal Markers

**FIGURE 3:**
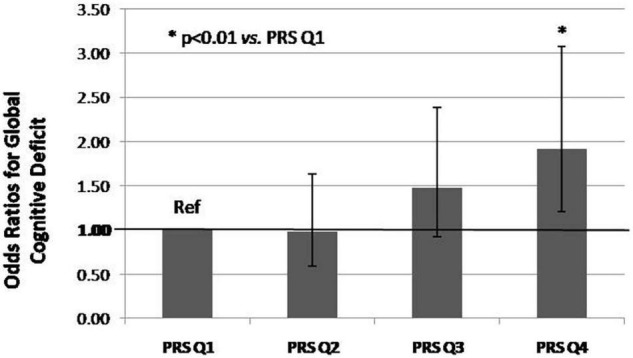
Odd ratios for PD prodromal marker Global Cognitive Deficit by PRS quartile (Q), com-pared to lower quartile in older population (*n* = 1,120). *P*-values derived from logistic regression models adjusted for MDSC1, MDSC2, age and sex. *P* for trend = 0.002. Q4 vs. Q1: OR = 1.923, 95%CI: 1.202–3.076, *p* = 0.006.

Results of multi-adjusted logistic regression models (adjusted for MDSC1, MDSC2, age and sex) investigating the association between PRS and each prodromal PD marker revealed significant association between PRS and global cognitive deficit exclusively (*p* = 0.003). Increases in PRS were associated with increased odds of having global cognitive deficit (OR = 1.021, 95%CI: 1.007–1.035, *p* = 0.003). Therefore, those in the higher quartile of PRS had higher odds of having global cognitive deficit, compared to participants in the lower quartile of PRS (OR = 1.923, 95%CI: 1.202–3.076, *p* = 0.006, Figure 3).

### Sensitivity Analysis

Excluding participants with dementia (*n* = 39) or additionally removing participants with MCI (*n* = 143) associations of PRS with global cognitive deficit remained unchanged, i.e., increases in PRS was associated with increased odds of having global cognitive deficit (OR = 1.023, 95%CI: 1.008–1.038, *p* = 0.002; OR = 1.025, 95%CI: 1.007–1.044, *p* = 0.007; respectively), while those in the higher quartile of PRS had higher odds of having global cognitive deficit, compared to participants in the lower quartile of PRS (excluding participants with dementia: OR = 1.990, 95%CI: 1.209–3.277, *p* = 0.007; additionally removing participants with MCI: OR = 2.060, 95%CI: 1.123–3.778, *p* = 0.020).

Increases in PRS were associated with lower individual cognitive domain z scores (memory: *b* = −0.005, 95%CI: −0.008 to −0.001, *p* = 0.020, language: *b* = −0.005, 95%CI: −0.009 to −0.001, *p* = 0.015, attention-speed: *b* = −0.010, 95%CI: −0.016 to −0.005, *p* < 0.001, executive: *b* = −0.004, 95%CI: −0.007 to −0.000, *p* = 0.045) with the exception of visual-spatial functioning (*b* = −0.003, 95%CI: −0.008 to 0.001, *p* = 0.169). Including all cognitive domains z scores in the same model, PRS was negatively associated with attention-speed z scores (*p* = 0.012).

Increases in PRS were associated with higher odds of language and attention-speed deficits (OR = 1.015, 95%CI: 1.002–1.027, *p* = 0.025; OR = 1.013, 95%CI: 1.000–1.026, *p* = 0.042; respectively). When including deficits of all cognitive domains in the same model, PRS was not associated with any of them (*p* > 0.05), suggesting associative dependency.

When we excluded the prodromal marker of global cognitive deficit from pPD probability calculation, the associations of PRS with increased odds of having 30% or 50% or more pPD probability remained unchanged (OR = 1.031, 95%CI: 1.006–1.056, *p* = 0.013; OR = 1.048, 95%CI: 1.012–1.085, *p* = 0.009; respectively).

When we excluded the risk marker of having or not first-degree relative with PD from the calculation of pPD probability, results also remained unchanged, i.e., there were no significant associations of PRS with pPD probability (continuous variable) or total LR for risk markers (*p* > 0.05), while increases in PRS were associated with increased odds of having 30% or 50% or more pPD probability (OR = 1.031, 95%CI: 1.007–1.056, *p* = 0.010; OR = 1.052, 95%CI: 1.014–1.091, *p* = 0.006; respectively).

In addition, we performed the analysis splitting the sample according to whether they reported having first-degree relative with PD. In those having first-degree relative with PD (*n* = 43), we found that PRS was associated with increased pPD probability (continuous and the cut-off of 30% (*n* = 9), b = 0.017, 95%CI: 0.004–0.030, *p* = 0.013 and OR = 1.125, 95%CI: 1.011–1.253, *p* = 0.031; respectively), total LR for prodromal markers (*b* = 0.023, 95%CI: 0.007–0.039, *p* = 0.007) and higher odds of subthreshold Parkinsonism (OR = 1.257, 95%CI: 1.018–1.550, *p* = 0.033). On the other hand, in those had not first-degree relative with PD (*n* = 1074), PRS was associated with higher odds of having 30% or more pPD probability (OR = 1.024, 95%CI: 1.000–1.050, *p* = 0.050), 50% or more pPD probability (OR = 1.040, 95%CI: 1.002–1.081, *p* = 0.041) and global cognitive deficit (OR = 1.020, 95%CI: 1.006–1.034, *p* = 0.005).

Finally, when we used PRS higher quartile as a pPD risk marker, pPD probability was significantly lower than when we used the presence of first-degree relative with PD [2.50 (1.00, 6.63) vs. 2.74 (1.14, 7.08), *p* = 0.007]. However, this difference was not significant in those who had not first-degree relative with PD (*p* = 0.694), since 50% of this sample received the same LR (1.00), while 25% received greater (1.57) and 25% lower LR (0.45) when PRS higher quartile was served as a marker instead of the marker of presence of first-degree relative with PD.

## Discussion

In the present study, we explored the effect of genes on prodromal PD by screening and integrating PD- associated SNPs identified from large GWASs and building polygenic risk models. Such PRS reflect the cumulative genome-wide impact of common genetic variation on a given phenotype into a single measure of genetic risk. We used a large population-based cohort which enabled us to obtain more stable effect size estimates and better risk predictions. Several PRS models containing different numbers of SNPs were built for investigating possible associations with probability of pPD and its risk markers. A PRS model based on 90 SNPs (SNPs with a threshold of *p* < 5 × 10**^–^**^8^ from a large GWASs meta-analysis showed a significant association with pPD. Variants that do not reach GWAS significance do not seem to contribute to the prediction accuracy of pPD in our study.

Previous studies have shown that PRSs differentiated individuals already diagnosed with PD from unaffected individuals and several polygenic analysis have become standard tools for dissecting risk for polygenic disorders and related traits (Nalls et al., 2014). However, there are no studies that examine the relationship between PRS and pPD or with several prodromal markers. The results of our study suggest that PRS may further improve PD risk prediction. Hence, PRS can serve as a promising clinical tool in early screening and identifying at -risk asymptomatic individuals for disease prevention. PRSs could also lead to the reclassification of individuals more accurately into appropriate disease risk categories.

Furthermore, we found a significant correlation between PRS and global cognitive deficit, a prodromal marker of PD. However, we failed to detect other prodromal or risk markers associated with PRS. Additionally, the total LRs of risk markers was not associated with PRS.

This could be partly explained by the fact that several putative risk/protective factors for PD were not included in our analysis. In the HELIAD study, we had information on 13 of 17 MDS markers (eight of 10 prodromal) (Maraki et al., 2019a). Several risk assessments were based on questionnaires and we did not perform any specific tests for certain markers such as polysomnography for RBD, specific smell identification tests for olfactory dysfunction or questionnaires like Gastrointestinal Dysfunction Scale for Parkinson’s Disease (GIDS-PD) to quantitatively assess features of gastrointestinal dysfunction (GID) symptoms. Moreover, MDS pPD criteria are still undergoing validation and each criterion may not specific to pPD or may overlap with other neurodegenerative diseases. Therefore, although we have considered several comorbidities we cannot exclude or calculate the role of others.

For instance, olfactory dysfunction is one of the markers not included in our analysis. Olfactory dysfunction is common in Parkinson’s disease (PD) and often predates the diagnosis by years, reflecting early deposition of Lewy pathology, the histologic hallmark of PD, in the olfactory bulb (Fullard et al., 2017). Olfactory function is also correlated with other non-motor features of PD and may serve as a predictor of cognitive decline. Hyposmia/anosmia is often seen in SNCA, GBA and LRRK2 carriers. However, it has variable penetrance in both sporadic and monogenic PD (Chase and Markopoulou, 2020). Therefore, while the hyposmia has been implicated as a “genetic” marker of PD in familial cases it has not been evaluated in our cohort concealing a possible association.

In an effort to explain this significant association of PRS with global cognitive deficit in pPD, we present previous studies showing how cognitive function influences the risk of parkinsonism and the genetic basis of cognition in PD.

Cognitive impairment and dementia are well established disorders in PD. As many as 80% of patients who are alive, 10 years after diagnosis are expected to develop dementia (Davis and Racette, 2016; Aarsland et al., 2017; Darweesh et al., 2017). Based on these findings global cognitive deficit was included in the calculation of risk markers (Heinzel et al., 2019a).

We have also recently reported results from HELIAD study investigating associations between pPD probability and cognitive function showing that higher probability of pPD was associated with lower cognitive performance in all domains. This may reflect a widespread pathologic process and non-dopaminergic pathways involved in neurodegeneration (Bougea et al., 2019).

As far as the genetic background of cognitive deficit concerns, most of the patient cohorts investigating cognitive impairment in PD have used candidate gene approaches so far. For instance, GBA, one of the most important genetic factors for PD, has also been implicated in cognitive impairment in PD (Alcalay et al., 2012; Mata et al., 2016; D’Souza and Rajkumar, 2020). Other studies have assessed the role of LRRK2, MAPT and SNCA for dementia in PD but results have often been inconclusive or not replicated independently (Mata et al., 2014). There were few studies so far that linked the cumulative burden of PD genetic risk factors with patient’s cognitive deficit. Thus, a recent study showed that PRS was associated with significantly faster cognitive decline (Paul et al., 2018).

However, the main question, as far as cognitive decline in PD is concerned, is whether this association is driven by phenotypic and pathogenetic links with other neurodegenerative disorders. The sensitivity analysis that we performed, excluding participants with dementia and MCI, enforces the implication of an independent association. Therefore, this significant association between PRS and cognitive deficit is attributed solely to cognitive decline in the context of PD.

The assumption of a distinct genetic component of cognition in PD is supported by a number of studies investigating phenotypic and genotypic links between PD and other associated disorders. The Brainstorm Consortium, collaboration among GWAS meta-analysis consortia for 25 disorders, performed a comprehensive correlation analysis of brain disorders. Neurological disorders showed a limited extend of genetic correlation suggesting greater diagnostic specificity and more distinct etiologies (Brainstorm et al., 2018). The absence of genetic overlap between AD and PD reported that might be indicative of different biological pathways solidifies our findings.

In terms of the distinct cognitive impairment profile detected in PD, there are a number of studies showing that cognitive impairment involves executive, attention visuospatial and memory impairment with the language being usually preserved (Fang et al., 2020). Executive dysfunction is at the root of most cognitive changes in PD while the attentional deficit is always present and has been shown to interfere significantly in the patients’ quality of life (Jalakas et al., 2019). Our findings are in consistent with this distinct clinical characteristics and PRS has been associated with them. Attention speed deficit was the most significantly associated cognitive domain with PRS. Declining processing speed in PD seems to have structural correlates with cortical thinning in temporoparietal regions, changes in diffusion MRI, especially in the cingulum tract, and decreased functional connectivity in posterior brain networks (Jalakas et al., 2019). All this evidence highlights even further these interesting associations that we detected at a prodromal stage.

A robust argument that could explain the association of PRS with cognitive deficit in pPD is that many genes that are included in PRS such as GBA, SNCA and MAPT, have been linked with cognitive outcomes in PD separately. For instance, results from the more recent large studies including more than a thousand subjects indicate an effect of SNCA variability on cognitive decline in PD (Guella et al., 2016). *GBA* status has been identified as one of the primary biological factors associated with cognitive status (Phongpreecha et al., 2020). *GBA* carriers had worse performance across most cognitive measures and the effect of this genetic factor on cognitive decline has been highlighted in many studies so far (D’Souza and Rajkumar, 2020). Previous reports on *MAPT* and cognition are mixed, with some studies reporting faster decline in MMSE scores and greater dementia risk in PD patients with the H1 haplotype and others showing a greater association between the H1 haplotype and PD diagnosis among those with dementia (Williams-Gray et al., 2009; Seto-Salvia et al., 2011).

Although evidence supporting an influence of genetics on cognition in PD is beginning to accumulate, the existing studies present variable methodology, conflicting results and limited number of candidate genes. High quality standardized data on cognition is also lacking in most studies. Our study is the only population based prospective study to investigate PRS with many prodromal markers of Parkinson’s disease and to highlight a significant association between the accumulation of many common genetic variants and cognitive deficits. The identification of the genetic influence on prodromal phase is even more robust especially when certain criteria on prodromal disorders are well established. This procedure allows the detection of a pure association between multiple domains of the disorder and the genetic susceptibility without the interaction of other clinical features.

## Data Availability Statement

The original contributions presented in the study are included in the article/supplementary material, further inquiries can be directed to the corresponding author/s.

## Ethics Statement

The studies involving human participants were reviewed and approved by institutional review boards (University of Thessaly, Larisa, Greece, and the National and Kapodistrian University of Athens, Greece Ethics Committees). The patients/participants provided their written informed consent to participate in this study.

## Author Contributions

MM, NS, and GX: conceptualization. MM, AH, NM, LS, MS, NS, and GX: methodology. AH and NM: software. MM, AH, and NM: formal analysis and data curation. MY, MK, ED, GH, PS, NS, and GX: investigation and resources. MM and GX: writing—original draft preparation. AH, NM, MY, LS, and NS: writing—review and editing. GX and NS: supervision. MY, MK, ED, GH, NS, and GX: project administration. NS and GX: funding acquisition. All authors have read and agreed to the published version of the manuscript.

## Conflict of Interest

The authors declare that the research was conducted in the absence of any commercial or financial relationships that could be construed as a potential conflict of interest.

## Publisher’s Note

All claims expressed in this article are solely those of the authors and do not necessarily represent those of their affiliated organizations, or those of the publisher, the editors and the reviewers. Any product that may be evaluated in this article, or claim that may be made by its manufacturer, is not guaranteed or endorsed by the publisher.

## Footnotes

1
http://prsice.info/

## References

[B1] Aarsland D. Creese B. Politis M. Chaudhuri K. R. Ffytche D. H. Weintraub D. (2017). Cognitive decline in Parkinson disease. *Nat. Rev. Neurol.* 13 217–231. 28257128 10.1038/nrneurol.2017.27 PMC5643027

[B2] Abraham G. Qiu Y. Inouye M. (2017). FlashPCA2: principal component analysis of Biobank-scale genotype datasets. *Bioinformatics* 33 2776–2778. 10.1093/bioinformatics/btx299 28475694

[B3] Alcalay R. N. Caccappolo E. Mejia-Santana H. Tang M. Rosado L. Orbe Reilly M. (2012). Cognitive performance of GBA mutation carriers with early-onset PD: the CORE-PD study. *Neurology* 78 1434–1440. 10.1212/WNL.0b013e318253d54b 22442429 PMC3345785

[B4] American Psychiatric Association [APA] (2000). *Diagnostic and Statistical Manual of Mental Disorders*, 4th Edn. Washington: American Psychiatric Publishing.

[B5] Anastasiou C. A. Yannakoulia M. Kosmidis M. H. Dardiotis E. Hadjigeorgiou G. M. Sakka P. (2017). Mediterranean diet and cognitive health: initial results from the Hellenic Longitudinal Investigation of Ageing and Diet. *PLoS One* 12:e0182048. 10.1371/journal.pone.0182048 28763509 PMC5538737

[B6] Berg D. Postuma R. B. Adler C. H. Bloem B. R. Chan P. Dubois B. (2015). MDS research criteria for prodromal Parkinson’s disease. *Mov. Disord.* 30 1600–1611. 10.1002/mds.26431 26474317

[B7] Berg D. Postuma R. B. Bloem B. Chan P. Dubois B. Gasser T. (2014). Time to redefine PD? Introductory statement of the MDS Task Force on the definition of Parkinson’s disease. *Mov. Disord.* 29 454–462. 24619848 10.1002/mds.25844 PMC4204150

[B8] Blessed G. Tomlinson B. E. Roth M. (1968). The association between quantitative measures of dementia and of senile change in the cerebral grey matter of elderly subjects. *Br. J. Psychiatry* 114 797–811. 10.1192/bjp.114.512.797 5662937

[B9] Bougea A. Maraki M. I. Yannakoulia M. Stamelou M. Xiromerisiou G. Kosmidis M. H. (2019). Higher probability of prodromal Parkinson disease is related to lower cognitive performance. *Neurology* 92 e2261–e2272. 10.1212/WNL.0000000000007453 30944240

[B10] Brainstorm C. Anttila V. Bulik-Sullivan B. Finucane H. K. Walters R. K. Bras J. (2018). Analysis of shared heritability in common disorders of the brain. *Science* 360:eaa8757. 10.1126/science.aap8757 29930110 PMC6097237

[B11] Chang C. C. (2020). Data Management and Summary Statistics with PLINK. *Methods Mol. Biol.* 2090 49–65. 10.1007/978-1-0716-0199-0_3 31975163

[B12] Chang C. C. Chow C. C. Tellier L. C. Vattikuti S. Purcell S. M. Lee J. J. (2015). Second-generation PLINK: rising to the challenge of larger and richer datasets. *Gigascience* 4:7. 10.1186/s13742-015-0047-8 25722852 PMC4342193

[B13] Chase B. A. Markopoulou K. (2020). Olfactory Dysfunction in Familial and Sporadic Parkinson’s Disease. *Front. Neurol.* 11:447. 10.3389/fneur.2020.00447 32547477 PMC7273509

[B14] Choi S. W. O’Reilly P. F. (2019). PRSice-2: polygenic Risk Score software for biobank-scale data. *Gigascience* 8:giz082. 10.1093/gigascience/giz082 31307061 PMC6629542

[B15] Cummings J. L. (1997). The Neuropsychiatric Inventory: assessing psychopathology in dementia patients. *Neurology* 48 S10–S16. 10.1212/wnl.48.5_suppl_6.10s 9153155

[B16] Dardiotis E. Kosmidis M. H. Yannakoulia M. Hadjigeorgiou G. M. Scarmeas N. (2014). The Hellenic Longitudinal Investigation of Aging and Diet (HELIAD): rationale, study design, and cohort description. *Neuroepidemiology* 43 9–14. 10.1159/000362723 24993387

[B17] Darweesh S. K. L. Wolters F. J. Postuma R. B. Stricker B. H. Hofman A. Koudstaal P. J. (2017). Association Between Poor Cognitive Functioning and Risk of Incident Parkinsonism: the Rotterdam Study. *JAMA Neurol.* 74 1431–1438. 10.1001/jamaneurol.2017.2248 28973176 PMC5822187

[B18] Das S. Forer L. Schonherr S. Sidore C. Locke A. E. Kwong A. (2016). Next-generation genotype imputation service and methods. *Nat. Genet.* 48 1284–1287. 10.1038/ng.3656 27571263 PMC5157836

[B19] Davis A. A. Racette B. (2016). Parkinson disease and cognitive impairment: five new things. *Neurol. Clin. Pract.* 6 452–458. 10.1212/CPJ.0000000000000285 27847686 PMC5100708

[B20] D’Souza T. Rajkumar A. P. (2020). Systematic review of genetic variants associated with cognitive impairment and depressive symptoms in Parkinson’s disease. *Acta Neuropsychiatr.* 32 10–22. 10.1017/neu.2019.28 31292011

[B21] Fahn S. Elton R. L. (1987). “Unified Parkinson’s disease rating scale,” in *Recent Developments in Parkinson’s Disease*, eds Fahn S. Marsden C. D. Calne D. B. Goldstein M. (Florham Park: Macmillan Healthcare Information), 153–163.

[B22] Fang C. Lv L. Mao S. Dong H. Liu B. (2020). Cognition Deficits in Parkinson’s Disease: mechanisms and Treatment. *Parkinsons Dis.* 2020:2076942. 10.1155/2020/2076942 32269747 PMC7128056

[B23] Fereshtehnejad S. M. Montplaisir J. Y. Pelletier A. Gagnon J. F. Berg D. Postuma R. B. (2017). Validation of the MDS research criteria for prodromal Parkinson’s disease: longitudinal assessment in a REM sleep behavior disorder (RBD) cohort. *Mov. Disord.* 32 865–873. 10.1002/mds.26989 28429825

[B24] Fountoulakis K. N. Tsolaki M. Iacovides A. Yesavage J. O’Hara R. Kazis A. (1999). The validation of the short form of the Geriatric Depression Scale (GDS) in Greece. *Aging* 11 367–372. 10.1007/BF03339814 10738851

[B25] Fullard M. E. Morley J. F. Duda J. E. (2017). Olfactory Dysfunction as an Early Biomarker in Parkinson’s Disease. *Neurosci. Bull.* 33 515–525. 10.1007/s12264-017-0170-x 28831680 PMC5636737

[B26] Giagkou N. Maraki M. I. Yannakoulia M. Kosmidis M. H. Dardiotis E. Hadjigeorgiou G. M. (2020). Prospective Validation of the Updated Movement Disorders Society Research Criteria for Prodromal Parkinson’s Disease. *Mov. Disord.* 35 1802–1809. 10.1002/mds.28145 32567751

[B27] Grove M. L. Yu B. Cochran B. J. Haritunians T. Bis J. C. Taylor K. D. (2013). Best practices and joint calling of the HumanExome BeadChip: the CHARGE Consortium. *PLoS One* 8:e68095. 10.1371/journal.pone.0068095 23874508 PMC3709915

[B28] Guella I. Evans D. M. Szu-Tu C. Nosova E. Bortnick S. F. Group S. C. S. (2016). Alpha-synuclein genetic variability: a biomarker for dementia in Parkinson disease. *Ann. Neurol.* 79 991–999. 10.1002/ana.24664 27091628

[B29] Hays R. D. Martin S. A. Sesti A. M. Spritzer K. L. (2005). Psychometric properties of the Medical Outcomes Study Sleep measure. *Sleep Med.* 6 41–44. 10.1016/j.sleep.2004.07.006 15680294

[B30] Heinzel S. Berg D. Gasser T. Chen H. Yao C. Postuma R. B. (2019a). Disease MDSTFotDoPs. Update of the MDS research criteria for prodromal Parkinson’s disease. *Mov. Disord.* 34 1464–1470. 31412427 10.1002/mds.27802

[B31] Heinzel S. Berg D. Gasser T. Chen H. Yao C. Postuma R. B. (2019b). Update of the MDS research criteria for prodromal Parkinson’s disease. *Mov. Disord.* 34 1464–1470. 10.1002/mds.27802 31412427

[B32] International Schizophrenia C. Purcell S. M. Wray N. R. Stone J. L. Visscher P. M. O’Donovan M. C. (2009). Common polygenic variation contributes to risk of schizophrenia and bipolar disorder. *Nature* 460 748–752. 10.1038/nature08185 19571811 PMC3912837

[B33] Jalakas M. Palmqvist S. Hall S. Svard D. Lindberg O. Pereira J. B. (2019). A quick test of cognitive speed can predict development of dementia in Parkinson’s disease. *Sci. Rep.* 9:15417. 10.1038/s41598-019-51505-1 31659172 PMC6817840

[B34] Karczewski K. J. Francioli L. C. Tiao G. Cummings B. B. Alfoldi J. Wang Q. (2020). The mutational constraint spectrum quantified from variation in 141,456 humans. *Nature* 581 434–443. 32461654 10.1038/s41586-020-2308-7 PMC7334197

[B35] Kosmidis M. H. Vlachos G. S. Anastasiou C. A. Yannakoulia M. Dardiotis E. Hadjigeorgiou G. (2018). Dementia Prevalence in Greece: the Hellenic Longitudinal Investigation of Aging and Diet (HELIAD). *Alzheimer Dis. Assoc. Disord.* 32 232–239. 10.1097/WAD.0000000000000249 29528855

[B36] Mahlknecht P. Gasperi A. Djamshidian A. Kiechl S. Stockner H. Willeit P. (2018). Performance of the Movement Disorders Society criteria for prodromal Parkinson’s disease: a population-based 10-year study. *Mov. Disord.* 33 405–413. 10.1002/mds.27281 29436728

[B37] Mahlknecht P. Gasperi A. Willeit P. Kiechl S. Stockner H. Willeit J. (2016). Prodromal Parkinson’s disease as defined per MDS research criteria in the general elderly community. *Mov. Disord.* 31 1405–1408. 10.1002/mds.26674 27273736

[B38] Maraki M. I. Stefanis L. Yannakoulia M. Kosmidis M. H. Xiromerisiou G. Dardiotis E. (2019a). Motor function and the probability of prodromal Parkinson’s disease in older adults. *Mov. Disord.* 34 1345–1353. 10.1002/mds.27792 31314148

[B39] Maraki M. I. Yannakoulia M. Stamelou M. Stefanis L. Xiromerisiou G. Kosmidis M. H. (2019b). Mediterranean diet adherence is related to reduced probability of prodromal Parkinson’s disease. *Mov. Disord.* 34 48–57. 10.1002/mds.27489 30306634

[B40] Marchini J. Howie B. Myers S. McVean G. Donnelly P. (2007). A new multipoint method for genome-wide association studies by imputation of genotypes. *Nat. Genet.* 39 906–913. 10.1038/ng2088 17572673

[B41] Mata I. F. Leverenz J. B. Weintraub D. Trojanowski J. Q. Chen-Plotkin A. Van Deerlin V. M. (2016). Variants are associated with a distinct pattern of cognitive deficits in Parkinson’s disease. *Mov. Disord.* 31 95–102. 10.1002/mds.26359 26296077 PMC4724255

[B42] Mata I. F. Leverenz J. B. Weintraub D. Trojanowski J. Q. Hurtig H. I. Van Deerlin V. M. (2014). MAPT, and SNCA genes and cognitive performance in Parkinson disease. *JAMA Neurol.* 71 1405–1412. 10.1001/jamaneurol.2014.1455 25178429 PMC4227942

[B43] McCarthy S. Das S. Kretzschmar W. Delaneau O. Wood A. R. Teumer A. (2016). A reference panel of 64,976 haplotypes for genotype imputation. *Nat. Genet.* 48 1279–1283. 10.1038/ng.3643 27548312 PMC5388176

[B44] McKeith I. G. Dickson D. W. Lowe J. Emre M. O’Brien J. T. Feldman H. (2005). Diagnosis and management of dementia with Lewy bodies: third report of the DLB Consortium. *Neurology* 65 1863–1872. 16237129 10.1212/01.wnl.0000187889.17253.b1

[B45] Michopoulos I. Douzenis A. Kalkavoura C. Christodoulou C. Michalopoulou P. Kalemi G. (2008). Hospital Anxiety and Depression Scale (HADS): validation in a Greek general hospital sample. *Ann. Gen. Psychiatry* 7:4. 18325093 10.1186/1744-859X-7-4 PMC2276214

[B46] Mirelman A. Saunders-Pullman R. Alcalay R. N. Shustak S. Thaler A. Gurevich T. (2018). Application of the Movement Disorder Society prodromal criteria in healthy G2019S-LRRK2 carriers. *Mov. Disord.* 33 966–973. 10.1002/mds.27342 29603409 PMC6105406

[B47] Nalls M. A. Blauwendraat C. Vallerga C. L. Heilbron K. Bandres-Ciga S. Chang D. (2019). Identification of novel risk loci, causal insights, and heritable risk for Parkinson’s disease: a meta-analysis of genome-wide association studies. *Lancet Neurol.* 18 1091–1102. 10.1016/S1474-4422(19)30320-5 31701892 PMC8422160

[B48] Nalls M. A. Pankratz N. Lill C. M. Do C. B. Hernandez D. G. Saad M. (2014). Large-scale meta-analysis of genome-wide association data identifies six new risk loci for Parkinson’s disease. *Nat. Genet.* 46 989–993. 10.1038/ng.3043 25064009 PMC4146673

[B49] Ntanasi E. Maraki M. Yannakoulia M. Stamelou M. Xiromerisiou G. Kosmidis M. H. (2020). Frailty and prodromal Parkinson’s disease: results from the HELIAD study. *J. Gerontol. A Biol. Sci. Med. Sci.* 76 622–629. 10.1093/gerona/glaa191 32761172

[B50] Ntanasi E. Yannakoulia M. Kosmidis M. H. Anastasiou C. A. Dardiotis E. Hadjigeorgiou G. (2017). Adherence to Mediterranean Diet and Frailty. *J. Am. Med. Dir. Assoc.* 19 315–322.e2. 29289542 10.1016/j.jamda.2017.11.005

[B51] Paul K. C. Schulz J. Bronstein J. M. Lill C. M. Ritz B. R. (2018). Association of Polygenic Risk Score With Cognitive Decline and Motor Progression in Parkinson Disease. *JAMA Neurol.* 75 360–366. 10.1001/jamaneurol.2017.4206 29340614 PMC5885856

[B52] Petersen R. C. Lopez O. Armstrong M. J. Getchius T. S. D. Ganguli M. Gloss D. (2018). Practice guideline update summary: mild cognitive impairment: report of the Guideline Development, Dissemination, and Implementation Subcommittee of the American Academy of Neurology. *Neurology* 90 126–135. 10.1212/wnl.0000000000004826 29282327 PMC5772157

[B53] Phongpreecha T. Cholerton B. Mata I. F. Zabetian C. P. Poston K. L. Aghaeepour N. (2020). Multivariate prediction of dementia in Parkinson’s disease. *NPJ Parkinsons Dis.* 6:20. 10.1038/s41531-020-00121-2 32885039 PMC7447766

[B54] Pilotto A. Heinzel S. Suenkel U. Lerche S. Brockmann K. Roeben B. (2017). Application of the movement disorder society prodromal Parkinson’s disease research criteria in 2 independent prospective cohorts. *Mov. Disord.* 32 1025–1034. 10.1002/mds.27035 28509336

[B55] Purcell S. Neale B. Todd-Brown K. Thomas L. Ferreira M. A. Bender D. (2007). PLINK: a tool set for whole-genome association and population-based linkage analyses. *Am. J. Hum. Genet.* 81 559–575. 10.1086/519795 17701901 PMC1950838

[B56] Seto-Salvia N. Clarimon J. Pagonabarraga J. Pascual-Sedano B. Campolongo A. Combarros O. (2011). Dementia risk in Parkinson disease: disentangling the role of MAPT haplotypes. *Arch. Neurol.* 68 359–364. 10.1001/archneurol.2011.17 21403021

[B57] Skorvanek M. Ladomirjakova Z. Han V. Lesko N. Feketeova E. Jarcuskova D. (2017). Prevalence of Prodromal Parkinson’s Disease as Defined by MDS Research Criteria among Elderly Patients Undergoing Colonoscopy. *J. Parkinsons Dis.* 7 481–489. 10.3233/JPD-161036 28387681

[B58] Tsapanou A. Gu Y. O’Shea D. M. Yannakoulia M. Kosmidis M. Dardiotis E. (2017). Sleep quality and duration in relation to memory in the elderly: initial results from the Hellenic Longitudinal Investigation of Aging and Diet. *Neurobiol. Learn. Mem.* 141 217–225. 10.1016/j.nlm.2017.04.011 28455107

[B59] Williams-Gray C. H. Evans J. R. Goris A. Foltynie T. Ban M. Robbins T. W. (2009). The distinct cognitive syndromes of Parkinson’s disease: 5 year follow-up of the CamPaIGN cohort. *Brain* 132 2958–2969. 10.1093/brain/awp245 19812213

